# Simultaneous determination of newly developed antiviral agents in pharmaceutical formulations by HPLC-DAD

**DOI:** 10.1186/s13065-016-0232-6

**Published:** 2017-01-03

**Authors:** Nourah Zoman Al-Zoman, Hadir Mohamed Maher, Amal Al-Subaie

**Affiliations:** 1 College of Pharmacy, Department of Pharmaceutical Chemistry, King Saud University, P.O. Box 22452, Riyadh, 11495 Saudi Arabia; 2 Faculty of Pharmacy, Department of Pharmaceutical Analytical Chemistry, University of Alexandria, El-Messalah, Alexandria, 21521 Egypt

**Keywords:** Antiviral agents, Viekira, HPLC-DAD, Ombitasvir, Paritaprevir, Ritonavir

## Abstract

**Background:**

Ombitasvir/paritaprevir/ritonavir/dasabuvir (Viekira Pak^®^) are the newest medicines approved for use in the treatment of hepatitis C virus (HCV) and are available in tablet form as an oral combination. Specifically, these agents are indicated in the treatment of HCV in patients with genotype 1 infection. Due to the therapeutic importance and increased use of Viekira Pak, proper methods for its determination in bulk and pharmaceutical formulations must be developed.

**Results:**

The present study describes the development and validation of a simple, rapid, selective and economical reverse phase high performance liquid chromatography-diode array detection (HPLC-DAD) method for the simultaneous determination of paritaprevir (PAR), ombitasvir (OMB), dasabuvir(DAS) and ritonavir (RIT) in bulk and pharmaceutical preparations. The proposed method was carried out using an RPC_18_ column (150 × 4.5 mm, 3.5 μ), with a mobile phase consisting of 10 mM phosphate buffer (pH 7)and acetonitrile (35:65, v/v) at a flow rate of 1 ml/min and a detection wavelength of 254 nm. Sorafenib (SOR) was selected as the internal standard to ensure that the quantitative performance was high. The method was validated based on its specificity, linearity, limit of detection, limit of quantitation, accuracy, precision, robustness and stability. The calibration curves for PAR, DAS, RIT and OMB were linear at 2.5–60, 1.25–30, 1.7–40 and 0.42–10 μg/ml, respectively, and all of the correlation coefficients were >0.999.

**Conclusions:**

The proposed method was successfully applied for the determination of ombitasvir/paritaprevir/ritonavir/dasabuvirin tablets, without interference from the excipient peaks. Hence, the method can be applied for the routine quality control analysis of the studied drugs, either in bulk or dosed forms.

**Graphical abstract:**
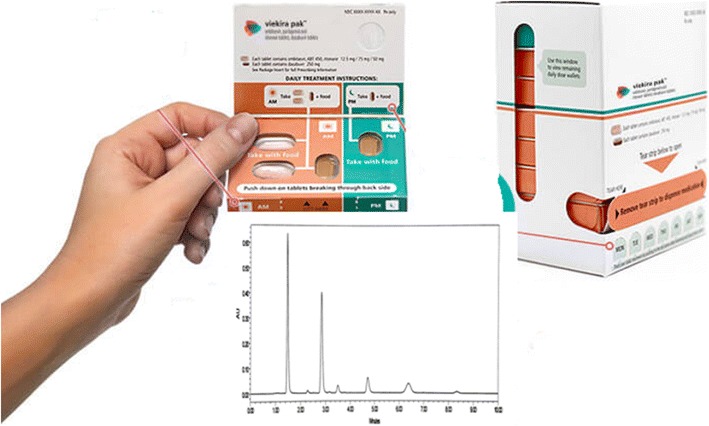
Simultaneous estimation of newly developed antiviral agents in pharmaceutical formulations by HPLC-DAD method

## Background

Approximately 180 million individuals worldwide are infected with chronic hepatitis C virus (HCV), and 500,000 patients die each year from liver disease associated with hepatitis C, making it the most common blood borne pathogen [1–3]. HCV, which belongs to the genus *Hepacivirus* within the family of *Flaviviridae*, is an enveloped virus with a single positive-stranded RNA genome [4]. In total, six different genotypes of HCV and multiple subtypes are known, and their distribution varies by region. In Saudi Arabia, HCV-genotype 4, followed by genotype 1, are the most prevalent [3, 5]. Increasing protective immune responses in human beings is difficult using classic approaches for virus control. As a result, an efficient vaccine for the prevention of HCV infection has not yet been developed, and the use of antiviral medications has been the only alternative considered for controlling the HCV epidemic [6]. In the past, a combination of peg-interferon (alfa-2a or alfa-2b) and ribavirin was the only available treatment regimen for HCV. However, these drugs have major disadvantages, such as long treatment courses, suboptimal efficacy, and/or harmful side effects. Therefore, the development of a new category of more potent and safer antiviral agents was required. Direct-acting antiviral (DAA) therapies, which were recently discovered and approved, offer good tolerability, short treatment duration, fewer side effects, and high cure rates. DAAs work by targeting a variety of stages in the HCV life cycle [6–11].

**Fig. 1:**
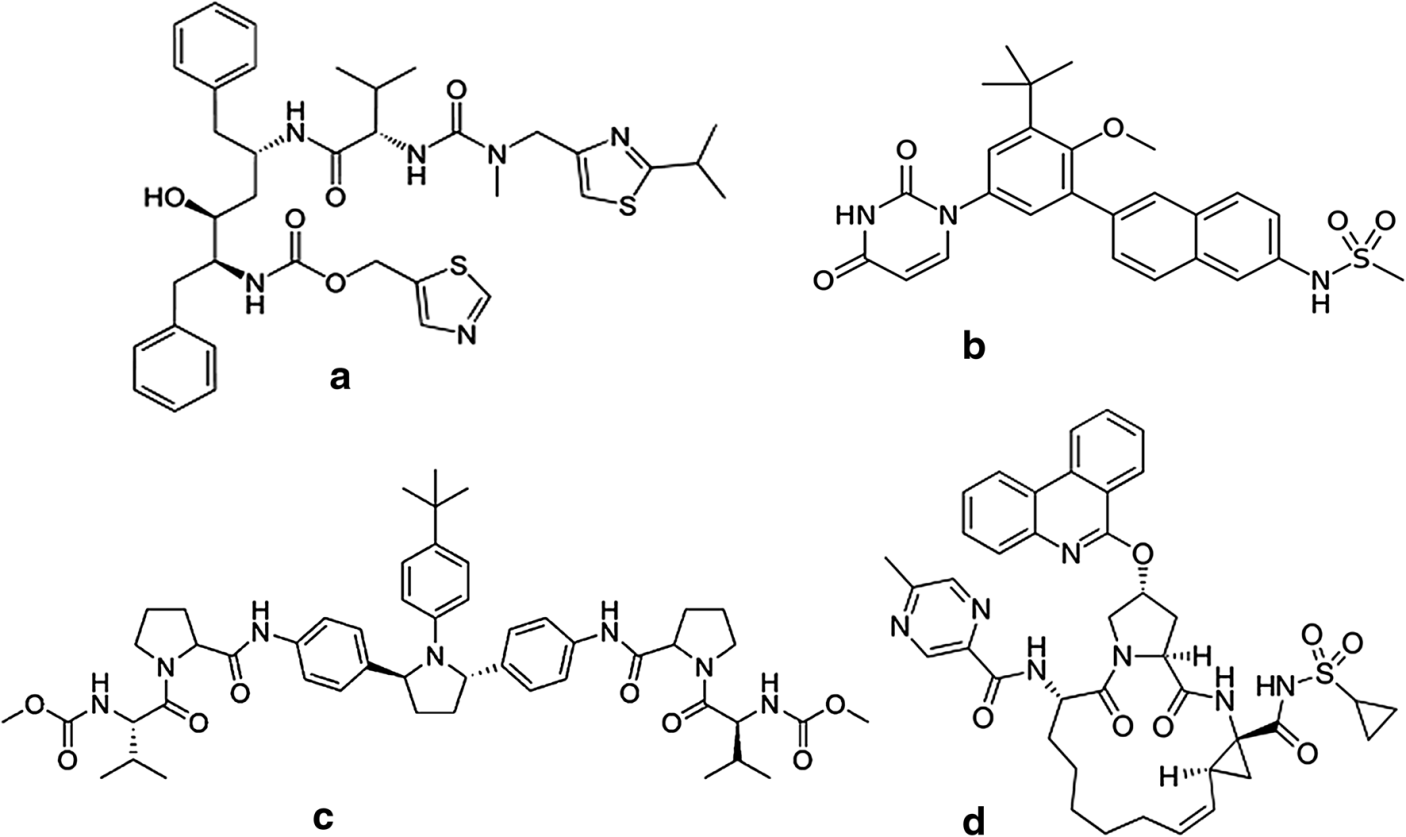
The chemical structures of the analytes in the present study: **a** ritonavir; **b** dasabuvir; **c** ombitasvir; **d** paritaprevir

On December 19, 2014, Viekira Pak^®^ (a combination of ombitasvir (OMB), paritaprevir (PAR) and ritonavir (RIT) tablets co-packaged with dasabuvir (DAS) tablets; Fig. 1) received FDA approval for the treatment of chronic HCV genotype 1 infection. Ombitasvir is a potent HCV NS5A inhibitor, paritaprevir is a potent inhibitor of NS3/4A protease, dasabuvir is a non-nucleoside NS5B polymerase inhibitor, and ritonavir is used as a pharmacokinetic enhancer for paritaprevir [12, 13]. Subsequently, Technivie^®^ has been approved by the FDA as the first DAA for the treatment of chronic HCV genotype 4 infections without requiring interferon co-administration. Technivie^®^ includes the same drugs as Viekira Pak^®^ with the exception ofdasabuvir [14].

A review of the literature revealed that CE [15, 16], HPLC [17–21], UPLC–MS/MS [22–24], LC–MS/MS [25, 26] and HPTLC [27, 28] methods have been reported for the analysis of RIT, individually or in combination with other drugs. However, a method for the simultaneous determination of OMB, PAR, RIT and DAS has not yet been reported. Therefore, the purpose of the present work was to develop a new method for the simultaneous determination of OMB, DAS, PAR and RIT in their bulk and pharmaceutical dosage forms. In this report, a simple, rapid, precise, accurate and selective RP-HPLC method was developed and validated in accordance with the international conference on harmonization (ICH) guidelines [29].

## Experimental

### Chemicals and reagents

OMB, DAS, PAR, RIT and internal standard SOR were purchased from Haoyuan Chemexpress Co., Ltd. (Shanghai, China). Samples of Viekirax^®^ and Exviera^®^tablets were obtained as gifts from King Faisal Specialist Hospital and Research Center (Riyadh, Saudi Arabia) and were manufactured by AbbVie Ltd. Acetonitrile (HPLC gradient-grade) was supplied by Panreac Quimica S.A. (Barcelona, Spain). Potassium dihydrogen orthophosphate anhydrous was obtained from WINLAB (Leicestershire, UK) and sodium hydroxide pellets were supplied by BDH Chemicals Ltd. (Poole, UK). Deionized water was used in all experiments.

### Instrumentation and chromatographic conditions

The HPLC system (Waters, Milford, MA, USA) consisted of a waters 1525 binary HPLC pump, a Waters 2998 Photodiode Array Detector, and a Waters 2707 Autosampler. The data were acquired and processed using Windows XP-based Waters Breeze 2 software. Ultrapure water (18 MΩ/cm) was produced by a Milli-Q^®^ Advantage A10^®^ Water Purification System (Billerica, MA, USA).

The chromatographic separations were carried out on a reverse phase Waters Symmetry^®^C_18_ column (150 × 4.5 mm i.d., particle size 3.5 μm). The mobile phase was a mixture of acetonitrile and 10 mM potassium dihydrogen orthophosphate (65:35, v/v; pH adjusted to 7 with sodium hydroxide) delivered at a flow rate of 1 ml/min. The mobile phase was filtered through 0.45-µm Whatman^®^filterpaper and sonicated for 20 min. Analysis was performed at ambient temperature, and the elution of the compounds was monitored by diode array detection (DAD) from 190 to 400 nm. The chromatograms were recorded at 254 nm, and the injection volume was 20 µl.

### Preparation of standard and sample solutions

#### Preparation of stock solutions

Accurate aliquots of 10 mg of PAR, RIT, OMB and internal standard SOR were each separately transferred into 10-ml volumetric flasks, dissolved using acetonitrile and diluted up to the mark with the same solvent to obtain primary stock solutions (concentration 1000 µg/ml) of each drug. The stock solution of DAS was prepared by weighing 10 mg of DAS and dissolving it in a very small amount of DMSO (10 drops); then, the final volume was achieved using acetonitrile to obtain a final concentration of 100 µg/ml.

Primary stock solutions of PAR, RIT, OM Band DAS were further diluted with the mobile phase to obtain working standards in the concentration range of 2.5–60, 1.25–30, 1.7–40 and 0.42–10 μg/ml for PAR, DAS, RIT and OMB, respectively. A standard concentration of 5 µg/ml SOR (internal standard) was added to the solutions.

### Preparation of sample solutions

Ten tablets of Viekirax^®^ (containing 75 mg PAR, 50 mg RIT and 12.5 mg OMB) were weighed and finely powdered. A quantity of the powder equivalent to 10 mg of PAR was weighed and transferred to a 10-ml volumetric flask. A small amount of acetonitrile was added to the flask, and the resulting mixture was sonicated for 20 min. The final volume was achieved using acetonitrile to obtain a final concentration of 1000 µg/ml of PAR. The solution was filtered through 0.45-µm filter paper (stock solution A).

Ten tablets of Exviera^®^tablet (containing 250 mg DAS) were powdered, and an amount equivalent to 10 mg of DAS was accurately weighed into a 10-ml volumetric flask and mixed with 10 drops of DMSO. A small amount of acetonitrile was then added to this flask. The solution was ultra sonicated for 20 min and filled with acetonitrile to obtain a final concentration of 1000 µg/ml of DAS. The solution was filtered through a 0.45-μm membrane filter (stock solution B).

Aliquots of sample stock solutions. A and B were further diluted with the mobile phase, and a constant amount of 5 μg/ml of SOR was added to each solution to obtain final concentrations of 40 μg/ml of PAR, 26.7 μg/ml of RIT, 6.7 μg/ml of OMB and 15 µg/ml of DAS. The resulting solutions were then subjected to analysis by the proposed HPLC method.

## Results and discussion

### Method development and optimization of chromatographic conditions

**Fig. 2:**
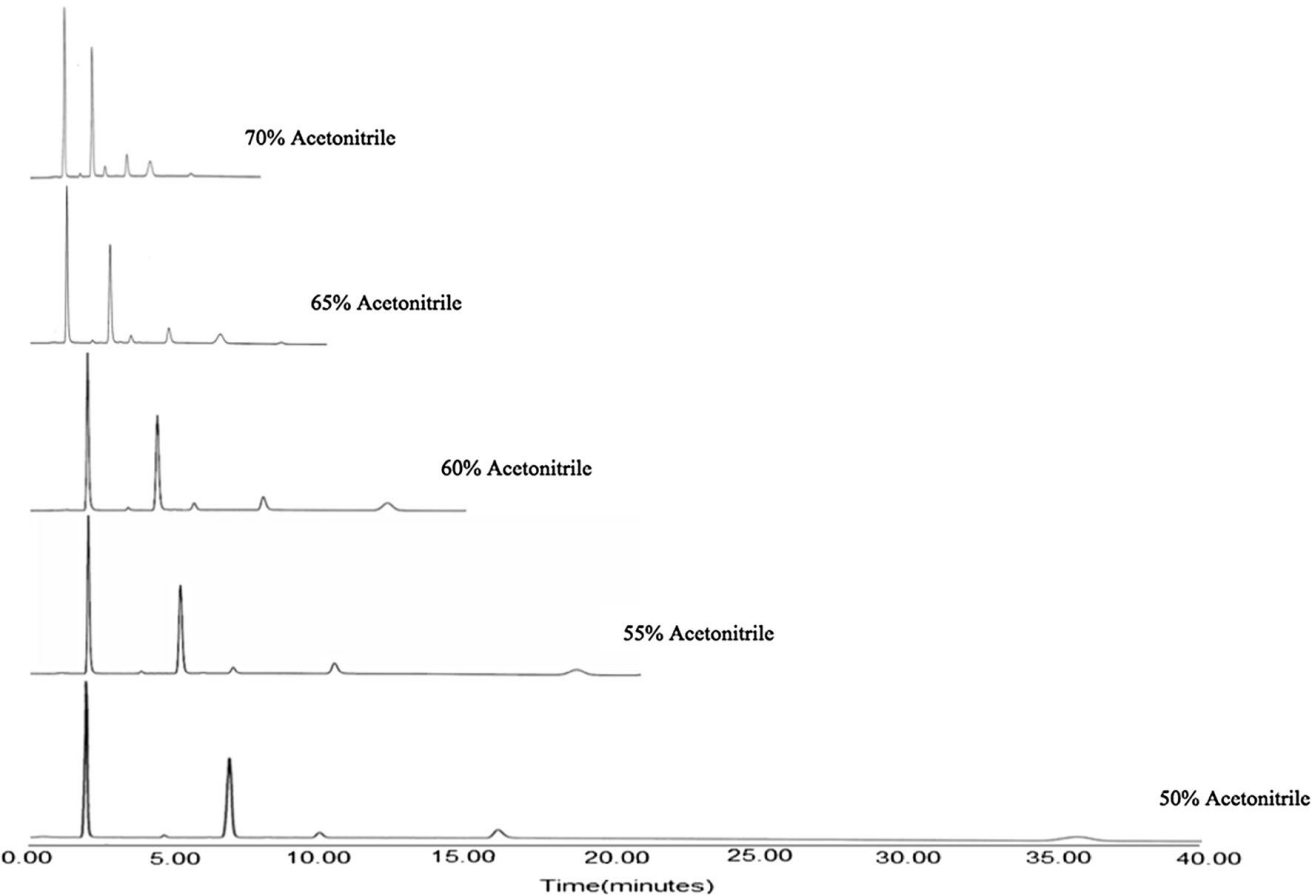
Effect of different ratios of acetonitrile in the mobile phase on the elution of the studied compounds, the order of elution is: paritaprevir (PAR), dasabuvir (DAS), ritonavir (RIT), and ombitasvir (OMB)

**Fig. 3:**
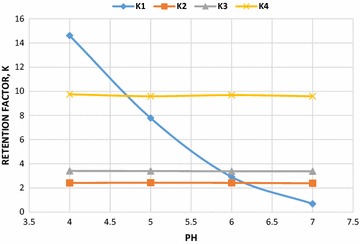
Effect of different ratios of acetonitrile in the mobile phase on the elution of the studied compounds, the order of elution is: paritaprevir (PAR), dasabuvir (DAS), ritonavir (RIT), and ombitasvir (OMB)

**Fig. 4:**
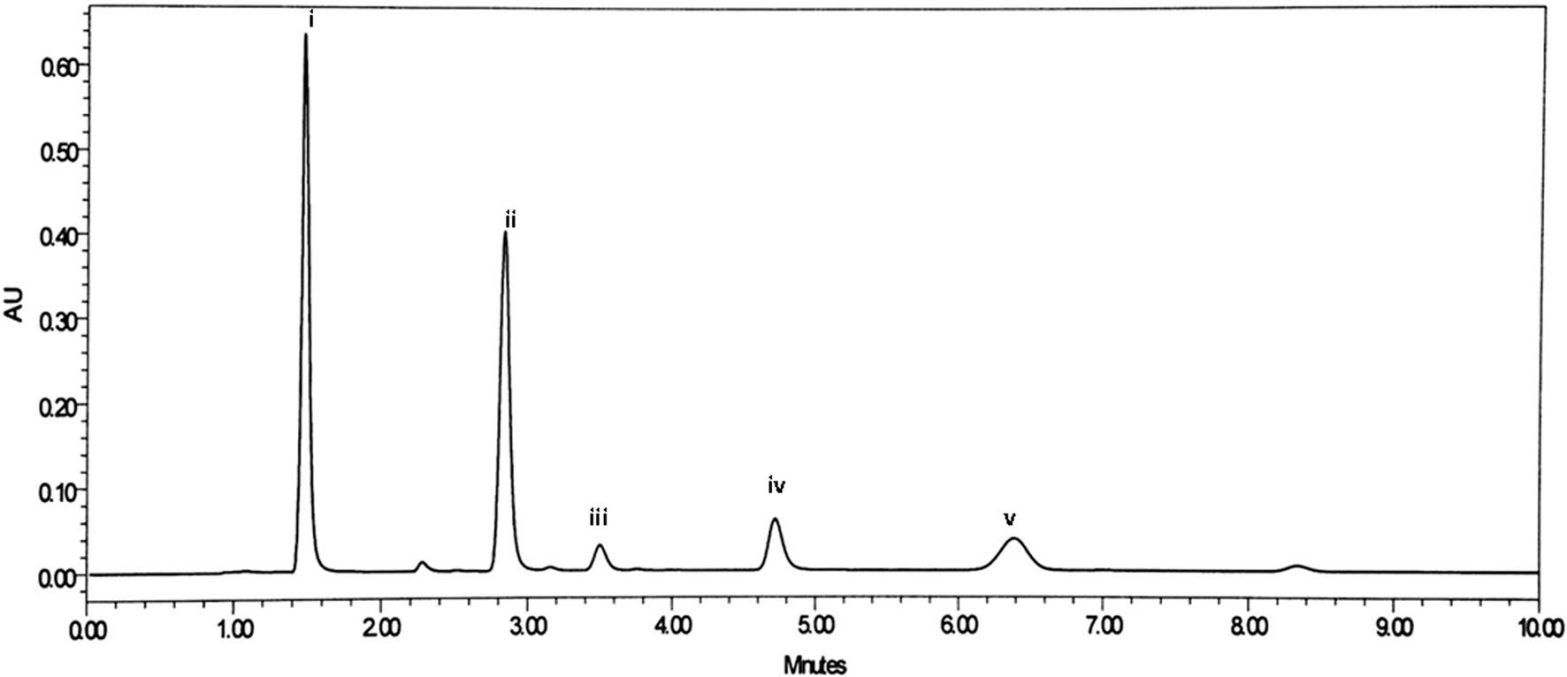
A typical chromatogram of mixed standard solution: (*i*) 40 μg/ml PAR; (*ii*) 15 μg/ml DAS; (*iii*) 26.7 μg/ml RIT; (*vi*) 5 μg/ml SOR (IS); (*v*) 6.7 μg/ml OMB

The method was developed based upon the experience obtained from the HPLC method previously developed for the analysis of RIT [17]. The previous experiment was performed using a mobile phase consisting of acetonitrile and phosphate buffer (pH 3) at a ratio of 60:40, v/v. For the separation of RIT from mixtures containing DAS, OMB and PAR, methanol and acetonitrile were used as organic modifier, peak symmetry and optimum pressure was obtained by using acetonitrile. Various ratios of acetonitrile and phosphate buffer solutions and different mobile phase pH values were tested using a C18 (150 × 4.5 mm, 3.5 μm) column, higher acetonitrile ratio resulted in shorter retention times of drugs Using this mobile phase ratio best results were obtained in terms of peak symmetry, selectivity and analysis time for drugs and the results are shown in Fig. 2. The pKa values of the studied drugs are reported in the literature as 2.8 for RIT, 2.5 for OMB, 4.6 for PAR, and 8.2 and 9.2 for DAS, which has two pKas. Therefore, the pH of the mobile phase was adjusted to 7 (Fig. 3). A wavelength of 254 nm was selected for the simultaneous determination of HVC drugs with high sensitivity. Moreover, the strength of the phosphate buffer solution (10–100 mM) was evaluated. Good resolution and reasonable retention times were observed for all of the drugs when acetonitrile:phosphatebuffer (0.01 M) (65: 35, v/v) was delivered at a flow rate of 1 ml/min (Fig. 4).

### Method validation

Validation of the optimized method was performed according to ICH Q2 (R1) guidelines [29]. The following validation characteristics were addressed: specificity, detection limit, quantitation limit, linearity, precision, accuracy and robustness.

### System suitability parameters

System suitability tests are used to verify that the resolution and reproducibility of the system are adequate. Several suitability parameters, including the capacity factor, selectivity, efficiency, resolution and tailing factor were calculated, as shown in Table 1. The peaks obtained were sharp and showed clear baseline separation.

**Table 1:** System suitability parameters for the determination of paritaprevir (PAR), dasabuvir (DAS), ritonavir (RIT), and ombitasvir (OMB) using the proposed HPLC method

Analyte	Retention time (min) (R_t_)	Capacity factor (k)	Selectivity (α)	Resolution (R_s_)	Tailing (T_f_)	Efficiency (no. of theoretical plates)
PAR	1.476	1.73	–	–	1	2443
DAS	2.835	4.13	2.39	10.62	1.20	8211
RIT	3.499	5.33	1.29	4.25	1	5555
SOR	4.720	7.53	1.41	6.49	1.20	10,085
OMB	6.388	10.53	1.40	6.64	1.20	6632

### Specificity

The specificity of the proposed HPLC method was assessed by comparing the spectrum of each drug in the sample with the reference drug spectrum using the diode array detector. Chromatograms obtained from standard solutions were also compared to those from the sample solutions, and interference was not observed in the presence of formulation excipients, indicating that the specificity of the method was satisfactory Table 5.

### Limit of detection (LOD) and limit of quantitation (LOQ)

The limit of detection and limit of quantitation were determined by diluting known concentrations of each drug until signal to noise ratios of approximately 3:1 and 10:1 were obtained, respectively. The LOD and LOQ of PAR, DAS, RIT and OMB, which represent the capability of the method to detect and quantify low concentrations, were 0.0024 and 0.0049 μg/ml, 0.00488 and 0.0098 μg/ml, 0.0521 and 0.1042 μg/ml, 0.0065 and 0.0130 μg/ml, respectively. This result indicates the capability of the method to detect and quantify low concentrations. The results are summarized in Table 2.

**Table 2:** Regression and statistical parameters for the determination of DAAs using the proposed HPLC method

	PAR	DAS	RIT	OMB
Linearity range (µg/ml)	2.5–60	1.25–30	1.7–40	0.42–10
LOD (µg/ml)^a^	0.0024	0.00488	0.0521	0.0065
LOQ (µg/ml)^b^	0.0049	0.0098	0.1042	0.0130
Intercept	0.0940	0.0525	0.0057	0.0124
Slope	0.1233	0.2122	0.0144	0.1725
Correlation coefficient (r)	0.9995	0.9998	0.9996	0.9997
S_a_^c^	0.0415	0.0230	0.0031	0.0137
S_b_^d^	0.0013	0.0015	0.0002	0.0084
S_y/x_^e^	0.0682	0.0378	0.0051	0.0016
F^f^	8441.8243	20320.4610	9008.7822	11307.0622
Significance F	8.4127E−08	1.4525E−08	7.3875E−08	4.6902E−08

Ombitasvir (OMB), paritaprevir (PAR), ritonavir (RIT), and dasabuvir (DAS)

^a^LOD: limit of detection

^b^LOQ: limit of quantitation

^c^S_a_: standard deviation of intercept

^d^S_b_: standard deviation of slope

^e^S_y/x_:standard deviation of residuals

^f^F: variance ratio, equals the mean of squares due to regression divided by the mean of squares about regression (due to residuals)

### Linearity

The linearity of the response of the detector for each drug was determined by plotting the response ratio (ratio of the peak area of the drug to that of the internal standard) versus the drug concentration and calculating the corresponding regression equation. The calibration curve was linear at concentrations of 2.5–60 µg/ml for PAR, 1.25–30 µg/ml for DAS, 1.7–40 µg/ml for RIT and 0.42–10 µg/ml for OMB. For all of the standard solutions, each concentration was injected in triplicate to obtain reproducible responses. According to the results of the regression analysis, which are given in Table 2, the method was linear, showing a correlation coefficient of >0.999.

The high values of the correlation coefficients (r) and negligible intercepts (a) indicated that the linearity of the calibration graphs was acceptable. Sy/x is a measure of the extent of deviation between the observed (measured) y-values and calculated y-values. For example, low values of Sy/x indicate that the point lies close to the regression line. The standard deviation (SD) of the intercept (Sa) and slope (Sb) were also calculated.

### Precision and accuracy

The repeatability of the developed method (intra and inter-day precision),which was expressed as the % RSD, and the accuracy, which was expressed as the %Er, were determined by injecting three different standard solution sat each of the low, medium and high concentration levels on the same day for the intra-day study (n = 3) and the following two consecutive days for the inter-day studies (n = 9). As shown in Table 3, the calculated % RSD and % Er were within the acceptable range of values, <2% for both % Er and % RSD.

**Table 3:** Intra-day and inter-day precision and accuracy for the determination of paritaprevir (PAR), dasabuvir (DAS), ritonavir (RIT), and ombitasvir (OMB) using the proposed HPLC method

Compound	Intraday precision and accuracy (n = 3)				Interday precision and accuracy (n = 9)		
	Standard concentration (μg/ml)	Mean % recovery ± SD	RSD (%)^a^	Er (%)^b^	Mean % recovery ± SD	RSD (%)^a^	Er (%)^b^
PAR	5	100.53 ± 0.599	0.596	−0.526	99.17 ± 1.227	1.237	0.831
	25	99.11 ± 0.133	0.134	0.893	99.22 ± 0.396	0.399	0.781
	50	101.39 ± 0.250	0.247	−1.387	101.29 ± 0.232	0.229	−1.288
DAS	3	99.72 ± 0.324	0.326	0.727	98.78 ± 0.567	0.574	1.223
	15	100.75 ± 0.143	0.142	−0.752	100.51 ± 0.271	0.270	−0.508
	25	100.38 ± 0.17	0.17	−0.384	100.45 ± 0.352	0.350	−0.451
RIT	3.3	101.19 ± 1.183	1.169	−1.191	101.32 ± 0.996	0.983	−1.316
	16.7	100.67 ± 0.069	0.068	−0.967	101.54 ± 0.466	0.459	−1.542
	33.3	100.92 ± 0.053	0.053	−0.925	101.32 ± 1.164	1.149	−1.315
OMB	0.83	99.23 ± 0.002	0.002	0.768	99.71 ± 1.612	1.617	0.291
	4.17	100.85 ± 0.001	0.001	−0.852	100.99 ± 0.312	0.309	−0.995
	8.33	101.64 ± 0.006	0.006	−1.637	101.66 ± 0.386	0.379	−1.659

^a^RSD (%): percentage relative standard deviation

^b^Er (%): percentage relative error

### Robustness

The robustness of the method was studied by making small but deliberate changes in the chromatographic parameters and evaluating the resulting % RSD. The optimal injection volume (±5 µl), detection wavelength (±1 nm), percentage of acetonitrile (±1 ml), mobile phase flow (±1 ml/min) and pH of the mobile phase (±0.1 unit) were studied by changing a single parameter while maintaining the others at a constant value. The % RSD is provided in Table 4. Significant changes in chromatograms were not observed % RSD < 2%, demonstrating the robustness of the developed method.

**Table 4:** Robustness of the proposed HPLC method

Parameter	PAR		DAS		RIT		OMB	
	RSD % of peak areas	k ± SD	RSD % of peak areas	k ± SD	RSD % of peak areas	k ± SD	RSD % of peak areas	k ± SD
Percentage of acetonitrile in the mobile phase [64, 65 and 66 ml]	0.290	0.48 ± 0.007	0.177	2.08 ± 0.005	0.446	2.81 ± 0.001	0.335	6.11 ± 0.002
pH of the aqueous phase [6.9, 7 and 7.1]	0.363	0.53 ± 0.011	0.178	2.03 ± 0.010	0.413	2.73 ± 0.001	0.471	5.85 ± 0.003
Flow rate [0.9, 1, and 1.1 ml/min]	0.239	0.53 ± 0.005	0.182	2.12 ± 0.005	0.32	2.87 ± 0.000	0.579	6.25 ± 0.003
Injection volume [15, 20, 25 µl]	0.255	0.53 ± 0.024	0.141	1.97 ± 0.026	0.80	2.68 ± 0.01	0.314	5.66 ± 0.007
Detection wavelength [253, 254 and 255 nm]	0.403	0.55 ± 0.007	0.089	1.99 ± 0.003	0.394	2.69 ± 0.001	0.580	5.75 ± 0.002

All results are average of three determinations

### Solution stability

The stabilities of both standard and sample solutions were examined, and changes in the corresponding chromatographs were not observed after 24 h at room temperature and over 3 weeks in a refrigerator (4 °C).

### Analysis of pharmaceutical formulations

The developed HPLC method with DAD was successfully applied to analyse the content of PAR, RIT and OMBin Viekirax^®^tablets and DAS in Exviera^®^tablets. Interfering peaks were not observed in the chromatogram of the marketed formulation, indicating that excipients used in the tablets did not interfere with the peaks of interest when the proposed method was employed. The mean % recovery of the drug content of the tablets was determined and was shown to range from 98 to 102%. The results are reported in Table 5.

**Table 5:** Assay of paritaprevir (PAR), dasabuvir (DAS), ritonavir (RIT) and ombitasvir (OMB) tablets by the proposed HPLC methods

Mean % recovery ± RSD^a^			
PAR	DAS	RIT	OMB
101.89 ± 0.264	99.61 ± 0.498	101.93 ± 0.862	102.28 ± 0.011

^a^Results are average of 6 experiments

## Conclusions

A new method based on isocratic RP-HPLC with DAD was developed and validated for the simultaneous determination of paritaprevir, ombitasvir, ritonavir and dasabuvir in bulk and pharmaceutical formulations. The reliability assessment showed that the proposed method was linear, accurate, precise, reproducible, specific and robust. Moreover, all four drugs were successfully resolved and quantified within a single analytical run with a short operating time (the elution time of the last peak was 6.3 min). Therefore, the developed method can be used in quality control studies, in which cost and time are concerning factors.

## Abbreviations

HPLC-DAD: high performance liquid chromatography-diode array detection ion
PAR: paritaprevir
OMB: ombitasvir
DAS: dasabuvir
RIT: ritonavir

## References

[1] Cooke GC, Lemoine M, Thursz M, Gore C, Swan T, Kamarulzaman A, DuCros P, Ford N (2013). Viral hepatitis and the global burden of disease: a need to regroup. J Viral Hepat.

[2] Berenguer M, López-Labrador FX, Wright TL (2001). Hepatitis C and liver transplantation. J Hepatol.

[3] World Health Organization (2005) Hepatitis C, Fact sheet 164, updated July 2015. http://www.who.int/mediacentre/factsheets/fs164/en/. Retrieved 3 Dec 2015

[4] Hoofnagle J (2002). Course and outcome of hepatitis C. Hepatol.

[5] Al-Faleh FZ, Huraib S, Sbeih F, Al-Karawi M, Al-Rashed R, Al-Mofleh IA, Sougiyyah M, Shaheen M, Ramia S (1995). Hepatitis C virus genotypes in patients with chronic liver disease and haemodialysis patients from Saudi Arabia. J Viral Hepat.

[6] Pawlotsky J (2014). New hepatitis C therapies: the toolbox, strategies, and challenges. Gastroenterology.

[7] Gutierrez J, Lawitz E, Poordad F (2015). Interferon-free, direct-acting antiviral therapy for chronic hepatitis C. J Viral Hepat.

[8] Jack K (2015). Advances in treatments for hepatitis C. Br J Health Care Manag.

[9] Majumdar A, Kitson MT, Roberts SK (2015). Treatment of hepatitis C in patients with cirrhosis: remaining challenges for direct-acting antiviral therapy. Drugs.

[10] Schinazi R, Halfon P, Marcellin P, Asselah T (2014). HCV direct-acting antiviral agents: the best interferon-free combinations. Liver Int.

[11] Zhang X (2016). Direct anti-HCV agents. Acta Pharm Sinica B.

[12] The medical letter on drugs and therapeutics. A 4-drug combination (Viekira Pak) for hepatitis C. J Am Med Assoc 2015;313: 1857–1858

[13] FDA, US food and drug administration. FDA approves Viekira Pak to treat hepatitis C.FDANews release, Dec. 19, 2014. http://www.fda.gov/NewsEvents/Newsroom/PressAnnouncements/ucm427530.htm. Retrieved 8 Dec 2015

[14] FDA, US food and drug administration. FDA approves Technivie for treatment of chronic hepatitis C genotype 4. FDA News release, 24 July 2015

[15] Gutleben W, Tuan ND, Stoiber H, Dierich MP, Sarcletti M, Zemann A (2001). Capillary electrophoretic separation of protease inhibitors used in human immunodeficiency virus therapy. J Chromatogr A.

[16] Breadmore MC, Theurillat R, Thormann W (2004). Determination of ribavirin in human serum and plasma by capillary electrophoresis. Electrophoresis.

[17] Rao BV, Vidyadhara S, Babu RR, Kumar BP, Kumar GK (2014). Analytical method development and validation for simultaneous estimation of lopinavir and ritonavir by RP-HPLC. Int J Res Dev Pharm L Sci.

[18] Jagadeeswaran M, Gopal N, Kumar KP, Kumar TS (2012). Quantitative estimation of lopinavir and ritonavir in tablets by RP-HPLC method. Pharm Anal Acta.

[19] Pabolu HK, Konidala SK (2013). New validated RP-HPLC method for the determination of ritonavir in bulk and pharmaceutical dosage form. Int J Pharm Pharm Sci.

[20] Kumar KV, Sudhakar M, Reddy YP, Malleshwari P, Hafeez SK (2014). RP-HPLC method development and validation for simultaneous estimation of lopinavir and ritonavir in dosage form and in plasma. Int J Pharm Res Rev.

[21] Sun H, Wang H, Ge X, Qin X (2011). Simultaneous determination of the combined drugs ribavirin and ceftriaxone sodium in human urine by HPLC-DAD. Int J Sci Innov Dis.

[22] Rezk MR, Basalious EB, Karim IA (2015). Development of a sensitive UPLC-ESI-MS/MS method for quantification of sofosbuvir and its metabolite, GS-331007, in human plasma: application to a bioequivalence study. J Pharm Biomed Anal.

[23] Shi X, Zhu D, Lou J, Zhu B, Hu AR, Gan D (2015). Evaluation of a rapid method for the simultaneous quantification of ribavirin, sofosbuvir and its metabolite in rat plasma by UPLC–MS/MS. J Chromatogr B Anal Technol Biomed Life Sci.

[24] Killi GD, Maddinapudi RK, Dinakaran SK (2014). A novel validated UPLC method for quantitation of lopinavir and ritonavir in bulk drug and pharmaceutical formulation with its impurities. Braz J Pharm Sci.

[25] Hendrikx JJ, Hillebrand MJ, Thijssen B, Rosing H, Schinkel AH, Schellens JH, Beijnen JH (2011). A sensitive combined assay for the quantification of paclitaxel, docetaxel and ritonavir in human plasma using liquid chromatography coupled with tandem mass spectrometry. J Chromatogr B Anal Technol Biomed Life Sci.

[26] Aouri M, Moradpour D, Cavassini M, Mercier T, Buclin T, Csajka C, Telenti A, Rauch A, Decosterd TA (2013). Multiplex liquid chromatography-tandem mass spectrometry assay for simultaneous therapeutic drug monitoring of ribavirin, boceprevir, and telaprevir. Antimicrob Agents Chemother.

[27] Abdelhay MH, Gazy AA, Shaalan RA, Ashour HK (2012). Validated stability-indicating HPLC and HPTLC methods for the determination of ritonavir in bulk powder and in capsules. J Food Drug Anal.

[28] Sadanshio PP, Wankhede SB, Chaudhari PD (2015). A validated stability indicating HPTLC method for estimation of ribavirin in capsule in presence of its alkaline hydrolysis degradation product. Anal Chem Lett.

[29] ICH Guideline (2005), Q2(R1): validation of analytical procedure: text and methodology. London

